# Quorum Sensing via Autoinducer‐2 Promotes Colonisation and Host Adaptation in *B. bifidum* PRL2010

**DOI:** 10.1111/1751-7915.70231

**Published:** 2025-09-15

**Authors:** Francesca Turroni, Chiara Tarracchini, Gabriele Andrea Lugli, Laura Maria Vergna, Giulia Alessandri, Sonia Mirjam Rizzo, Massimiliano G. Bianchi, Tom Coenye, Emanuele Selleri, Ovidio Bussolati, Douwe van Sinderen, Marco Ventura

**Affiliations:** ^1^ Department of Chemistry, Life Sciences, and Environmental Sustainability, Laboratory of Probiogenomics University of Parma Parma Italy; ^2^ Microbiome Research Hub University of Parma Parma Italy; ^3^ Department of Medicine and Surgery, Laboratory of General Pathology University of Parma Parma Italy; ^4^ Laboratory of Pharmaceutical Microbiology Ghent University Ghent Belgium; ^5^ APC Microbiome Institute and School of Microbiology Bioscience Institute, National University of Ireland Cork Ireland

**Keywords:** autoinducer‐2, bifidobacteria, *luxS*, quorum sensing

## Abstract

Autoinducer‐2 (AI‐2) is a key signalling molecule that in many bacteria facilitates interspecies communication by regulating gene expression in response to population density through a process known as quorum sensing. While this signalling mechanism has been extensively studied in Gram‐negative bacteria, its role in the genus *Bifidobacterium* remains poorly understood. In this study, an in silico analysis was conducted to examine the distribution of the *luxS* gene, which encodes the enzyme that synthesises the AI‐2 precursor, across *Bifidobacterium* genomes. Our analysis revealed that *luxS* is ubiquitously present in all publicly available bifidobacterial genomes. To explore the functional implications of *luxS*, gene expression profiling was performed on the model strain *B. bifidum* PRL2010 and its isogenic *luxS* insertion mutant, both grown in a medium simulating the human gut environment. RNA sequencing results indicated that disruption of *luxS* impairs the mutant strain's ability to (i) interact and communicate with the host, (ii) transport sugars, (iii) internalise potassium and iron, and (iv) cope with stress conditions. Collectively, these findings highlight the crucial role of AI‐2 in promoting colonisation and ensuring the persistence of PRL2010 within the competitive ecosystem of the human gut.

## Graphical abstract

Autoinducer‐2 (AI‐2), encoded by the *luxS* gene, is a molecule involved in the quorum sensing mechanism in bifidobacteria, typical intestinal bacteria. In this manuscript, the distribution of this gene in all bifidobacterial genomes deposited in the database has been studied. In addition, the role of this gene in the *Bifidobacterium bifidum* strain PRL2010—the reference strain for this species—was explored by gene inactivation experiments, revealing its involvement in metabolism, adhesion, and the ability to colonise the gut.

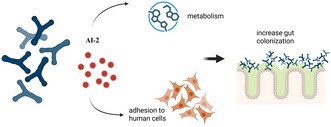

## 1 Introduction

Members of the genus *Bifidobacterium* are well‐established as predominant symbiotic microorganisms inhabiting the human gastrointestinal tract, where they are associated with a variety of host health‐promoting effects (Bottacini et al. 2017; Hidalgo‐Cantabrana et al. 2017; Alessandri et al. 2021; Ladeira et al. 2023; Martin et al. 2023). In this context, a growing body of scientific evidence highlights the capacity of bifidobacteria to modulate the development and function of the host immune system, while also playing a pivotal role in preserving intestinal eubiosis. This is achieved through mechanisms such as the inhibition of pathogenic microorganisms, enhancement of intestinal barrier integrity, and the prevention or attenuation of intestinal disorders such as inflammatory bowel disease (Tojo et al. 2014; Duranti et al. 2016; Ruiz et al. 2017; Alessandri, Milani, et al. 2019; Aw and Fukuda 2019; Shang et al. 2022), and various metabolic activities that have an impact on the physiology of the host (Milani et al. 2016; Alessandri, Ossiprandi, et al. 2019; Duranti et al. 2019; Tarracchini et al. 2021; Yin et al. 2023).

Due to their capacity for vertical transmission and their role as early colonisers of the human gut, bifidobacteria are thought to confer health benefits that commence immediately after birth (Milani et al. 2015; Duranti et al. 2017; Milani, Duranti, et al. 2017; Lin et al. 2022). Bifidobacteria exhibit their highest relative abundance and prevalence in the human gut during early life (0–6 months), a pattern particularly supported by host breastfeeding (Turroni, Peano, et al. 2012; Turroni et al. 2021; Yan et al. 2021; Lordan et al. 2024). Although their relative abundance significantly declines in parallel with the gut microbiota transition from an infant‐ to an adult‐like composition, bifidobacteria remain relatively stable and persist throughout the human lifespan, reaching also old age (Turroni et al. 2009; Kato et al. 2017; Longhi et al. 2024).

The persistence of *Bifidobacterium* species within the human intestinal environment is attributed to a range of specific genomic features that confer adaptive advantages. These include the production of diverse carbohydrate‐degrading enzymes, which facilitate their survival and competitiveness in the highly selective gut ecosystem, as well as the synthesis of extracellular structures, for example, pili, exopolysaccharides, and teichoic acids, which mediate interactions with and adhesion to the intestinal epithelium (Milani, Mangifesta, et al. 2017; Pyclik et al. 2020; Argentini et al. 2023; Tarracchini et al. 2023; Walsh et al. 2023; Lordan et al. 2024; Rizzo et al. 2024). However, bifidobacterial adhesion is unlikely to solely rely on these surface structures; rather, it appears to be the outcome of a complex, multifactorial process involving host‐derived, microbial, and environmental factors. Notably, quorum sensing—an intercellular communication mechanism based on population density‐dependent signalling—has emerged as a key regulatory factor influencing bifidobacterial gut colonisation and persistence (Waters and Bassler 2005; Atkinson and Williams 2009; Beurel 2024). This system operates through the accumulation of signalling molecules which, upon reaching a critical concentration, trigger a coordinated transcriptional response across the bacterial community. This response modulates a broad spectrum of physiological processes essential for bacterial survival, including genetic competence, virulence, motility, antibiotic production, biofilm formation, epithelial adhesion, acid tolerance, and stress adaptation (Xavier and Bassler 2003; Bassler and Losick 2006; Jin et al. 2012; Sun et al. 2014; Deng et al. 2020; Liu et al. 2021; Yu et al. 2022; Beurel 2024). Bifidobacteria are known to utilise autoinducer‐2 (AI‐2), which is a cell‐to‐cell interspecies universal signalling molecule whose production relies on the LuxS enzyme which catalyses the cleavage of S‐ribosyl‐homocysteine to homocysteine and 4,5‐dihydroxy‐2,3‐pentanedione, the latter representing the AI‐2 precursor (Xavier and Bassler 2003; Christiaen et al. 2014; Kareb and Aider 2020; Alessandri et al. 2021; Liu et al. 2021; Su and Ding 2023). Although a functional *luxS* gene has been identified in several *Bifidobacterium* species, only a limited number of studies have examined the effects of its inactivation. These investigations have demonstrated that *luxS* plays a role in biofilm formation and host colonisation in *Bifidobacterium longum* NCC2705, as well as in pathogen protection and, again, in intestinal colonisation in *Bifidobacterium breve* UCC2003 (Christiaen et al. 2014; Sun et al. 2014). In the present study, an in silico analysis was initially conducted to assess the distribution of the *luxS* gene across all currently recognised *Bifidobacterium* species, with the aim of expanding current knowledge regarding bifidobacterial LuxS. In addition, the potential for LuxS‐dependent AI‐2 activity in *Bifidobacterium bifidum* was investigated through a series of in vitro experiments using the model strain *B. bifidum* PRL2010.

## 2 Experimental Procedures

### 2.1 Distribution of *luxS* Homologues in Bifidobacterial Species

Amino acid sequences derived from both complete and draft genomes of all publicly available *Bifidobacterium* strains were retrieved from the National Center for Biotechnology Information (NCBI) database, comprising a total of 3359 bifidobacterial genomes at the time of analysis (May 2025). Subsequently, the deducted proteome of each publicly available bifidobacterial genome included in the present study was screened for the presence of *luxS* homologues, based on sequence similarity (cutoff e‐value < 1 × 10^−5^ and 60% identity over at least 80% of the protein sequence) to a custom reference database containing the LuxS protein sequence previously identified in *Bifidobacterium breve* UCC2003 (Christiaen et al. 2014). BlastP analysis was performed using DIAMOND software (Buchfink et al. 2015).

### 2.2 Bacterial Strains, Cultivation Conditions and Plasmids

*Bifidobacterium bifidum* PRL2010 and its isogenic *luxS*‐insertion mutant *B. bifidum* PRL2010 *luxS*::pFREM30 were routinely cultivated in modified de Man–Rogosa–Sharpe (MRS) medium without glucose, supplemented with 0.05% L‐cysteine hydrochloride and 2% lactose (mMRS) in an anaerobic chamber (concept 400, Ruskinn) at 37°C. For the cultivation of the mutant, mMRS was supplemented with 5 μg/mL chloramphenicol. *Escherichia coli* EC101, used as a host strain for the propagation of the aforementioned plasmid, was cultivated at 37°C in LB medium (Luria Bertani, Scharlau, Spain) supplemented with chloramphenicol at a final concentration of 25 μg/mL.

Plasmid pNZ003 was used as a positive control, while plasmid pFREM30, that is, a suicide vector, was employed to target *luxS* for mutagenesis by gene disruption through homologous recombination (Hoedt et al. 2021).

### 2.3 Construction of *B. bifidum* PRL2010 Insertion Mutant

General procedures for DNA manipulation were performed as previously described in the study in which the transformation protocol for *Bifidobacterium bifidum* PRL2010 was originally developed and optimised (Rizzo et al. 2024). Briefly, for the construction of plasmid pFREM30‐*luxS*, the to‐be‐targeted internal region of the gene (from base 61 to base 329 of the *luxS* gene) was amplified by PCR from chromosomal *B. bifidum* PRL2010 DNA employing the Q5 polymerase and primers LMV_57 (5′‐aatatagtgcacCCGTACGTGCGTTACATCGACAC‐3′) and LMV_58 (5′‐aatatatctagaAATTCCAGCGATTCCTTGAGGGC‐3′). Chromosomal DNA was extracted from *B. bifidum* PRL2010 cells using the GenElute Bacterial Genomic DNA kit (Merck, Germany) following the manufacturer's instructions. Plasmid DNA was isolated from *E. coli* EC101 using the GeneJET Plasmid Maxiprep Kit (Thermo Fisher Scientific, USA). The amplicon and plasmid were digested with ApaLI and XbaI, ligated by using the T4 DNA ligase, and introduced into *E. coli* EC101, as previously reported (Hanahan et al. 1991). To select transformants, the manipulated cells were plated on LB supplemented with 25 μg/mL chloramphenicol and the colonies were screened for the presence of the expected plasmid construct by colony PCR.

### 2.4 Preparation of Bifidobacterial Cells, Electroporation, and Selection of PRL2010 Mutants

An overnight culture of *B. bifidum* PRL2010 was inoculated into fresh mMRS broth supplemented with 7% (v/w) sucrose (sMRS) and cultivated at 37°C until the mid‐exponential growth phase (OD_600nm_ between 0.6 and 0.8). Subsequently, cells were harvested by centrifugation (4500 × *g* for 10 min at 4°C), washed twice with an ice‐cold citrate‐sucrose buffer (0.5 M sucrose, pH 5.8), and resuspended in 250 μL of the same buffer. Subsequently, cells were subjected to electroporation as previously described (Rizzo et al. 2024). After cell electroporation, bifidobacterial cells were resuspended in 950 μL of sMRS and incubated for 3 h at 37°C in an anaerobic cabinet. Then, cells were plated on sMRS agar supplemented with 5 μg/mL chloramphenicol and incubated anaerobically at 37°C for 48 h. Potential mutants were identified through colony PCR using the primers LMV_66 (5′‐CCCACGTGGCCTTGTATG‐3′) and LMV_65 (5′‐GAAAAGCCGATTGTCGAAAG‐3′), which annealed to the chromosomal gene outside the gene target region, and new_MCS1 (5′‐CGAATCGCCAACGTTTTC‐3′), which annealed to the (integrated) pFREM30 plasmid.

### 2.5 Detection of AI‐2 Production

The *Bifidobacterium* strains used for the AI‐2 biosensor assay, that is, *B. bifidum* PRL2010 wt and *luxS*::pFREM30 strains as well as *Bifidobacterium breve* 12 L, were cultured anaerobically at 37°C in modified Columbia Broth (mCol). The latter was prepared to contain galactose as the sole carbon source, with a final concentration of 1% w/v, to eliminate possible glucose traces since the latter has been reported to possibly interfere with AI‐2 detection by a biosensor assay (Bassler et al. 1993; Janssens et al. 2007; Christiaen et al. 2014; Kile et al. 2014). *Vibrio harveyi* BB170 biosensor was cultivated in Marine Broth at 30°C with agitation (Christiaen et al. 2014). A late exponential growth phase/early stationary phase culture of each selected *Bifidobacterium* strain was centrifuged twice at 5000 × *g* for 5 min at room temperature. The supernatant was then neutralised at pH 7 by using 5 M NaOH to exclude any possible interference with the detection of AI‐2 due to pH effects, as previously described (Taga 2005; Christiaen et al. 2014; Kile et al. 2014). Subsequently, the supernatants were filter‐sterilised and diluted to a final concentration of 20% (v/v) using sterile deionised milliQ water. Finally, an overnight culture of the reporter strain was diluted 1:5000 into fresh sterile Marine Broth. 100 μL of this cell suspension was aliquoted into a black 96‐well microtiter plate (Sarstedt, Germany) and mixed with 100 μL of each prepared cell free supernatants. The plate was incubated at 30°C and bioluminescence was measured every hour for a total of 5 h in a Tristar 3 microplate reader (TriStar^2^ LB 942; Berthold Technologies, Germany). AI‐2 activity was quantified as Relative Luminescence Units (RLU) at the time point when the negative control (mCol medium instead of culture supernatants) produced the lowest amount of luminescence. Supernatant of an overnight *V. harveyi* BB170 culture was used as a positive control (Taga 2005; Sun et al. 2014).

### 2.6 Mutant Genome Sequencing and Assembly

To assess whether plasmid insertion had occurred in the expected position within the *B. bifidum* PRL2010 genome, chromosomal DNA extracted from the mutant was subjected to whole‐genome sequencing on a MinION Mk1B sequencing platform (Oxford Nanopore Technologies, UK). DNA library preparation was performed using the Ligation sequencing gDNA—Native Barcoding Kit 24 V14 (Oxford Nanopore Technologies, UK), according to the manufacturer's instructions. In detail, approximately one μg of high molecular weight genomic DNA was used for library preparation, while for library cleanup the Long Fragment Buffer (LFB) was used to enrich long DNA fragments. The genomic DNA sample was barcoded and ligated to a specific adapter, while purification and quantification steps were performed involving the AMPure XP DNA purification beads (Oxford Nanopore Technologies, UK) and the fluorometric Qubit quantification system (Life Technologies, USA), respectively. Finally, the sample was loaded on a 2200 TapeStation instrument (Agilent Technologies, USA) to verify DNA fragment length. A final concentration of 300 μg was loaded on the instrument, following the manufacturer's instructions. Sequencing was performed on a MinION device (Oxford Nanopore Technologies, UK) using an R10.4.1 flow cell. The MinKNOW software was used to control the sequencing device, collect sequencing data, basecall, and demultiplex. At the same time, the mutant was also sequenced through the Illumina platform (Illumina, San Diego, USA). In this case, genome libraries were prepared using an Illumina Nextera XT DNA Library Preparation Kit (Illumina Inc., San Diego, USA). Libraries were quantified using a fluorometric Qubit quantification system (Life Technologies, USA), loaded on a 2200 TapeStation instrument (Agilent Technologies, USA), and normalised to 4 nM. Sequencing was performed using the Illumina MiSeq platform with a 600‐cycle flow cell version 3 (Illumina Inc., San Diego, USA). The obtained long reads were quality‐filtered using the Fitlong tool (https://github.com/rrwick/Filtlong), while short reads were filtered through the fastq‐mcf script (https://github.com/ExpressionAnalysis/ea‐utils). Subsequently, the filtered fastq file of MinION long reads, obtained from genome sequencing efforts, was used as input for genome assembly through CANU software (Koren et al. 2017). The resulting genome sequence was polished through Polypolish (Wick and Holt 2022) using Illumina paired‐end reads. The whole process was managed by the MEGAnnotator2 pipeline (Lugli et al. 2023). Then, the alignment tool MUMmer4 (Marcais et al. 2018) was employed to identify gaps between the assembled *B. bifidum* PRL2010 *luxS*::pFREM30 and *B. bifidum* PRL2010 wt genome sequences.

### 2.7 Mutant Stability

To assess the stability of the integrated pFREM30 plasmid, a spot assay was carried out, and the presence of the plasmid was checked by colony PCR. For this assay, *B. bifidum luxS*::pFREM30 was cultivated in mMRS broth and in Infant Gut Super Medium (IGSM) (Alessandri et al. 2022) with or without the supplementation of 5 μg/mL chloramphenicol. Every day, for a total of 2 weeks, an aliquot of the culture was sub‐cultured in fresh medium, and every second day, the overnight cultures of the two growth conditions were serially diluted and spotted on mMRS agar plates with or without 5 μg/mL chloramphenicol. Plates were incubated under anaerobic conditions at 37°C for 48 h and, subsequently, a colony PCR was performed on one colony per replicate with primers LMV_65 and LMV_66. The experiments were performed in triplicate.

### 2.8 Transcriptome Analysis of *B. bifidum* PRL2010 and *B. bifidum* PRL2010 *luxS* ::pFREM30 In Vitro Cultivated in a Growth Medium Simulating Human Intestinal Environment

To evaluate if the inactivation of *luxS* has any transcriptional impact on *B. bifidum* PRL2010, a comparison of the transcriptome of the insertion mutant *B. bifidum* PRL2010 *luxS*::pFREM30 with that obtained from *B. bifidum* PRL2010 wild‐type (wt) was performed. Specifically, the two strains were inoculated in a human gut environment–simulating culture medium for 8 h until the exponential growth phase (Alessandri et al. 2022). Cells were harvested by centrifugation at 5000 × *g* for 5 min when the mid‐exponential growth phase was reached, after which RNA extraction and sequencing were performed (see below for further details). Growth assays were carried out in triplicate.

#### 2.8.1 Prokaryotic RNA Extraction and Sequencing

Total RNA from each bifidobacterial culture was isolated as previously described (Turroni et al. 2016). Briefly, bifidobacterial cell pellets were resuspended in 1 mL of QIAzol lysis reagent (Qiagen, Germany) and transferred into a sterile tube containing glass beads. Cells were lysed by alternating 2 min of stirring the mix on a bead beater with 2 min of static cooling on ice. These steps were repeated three times. Lysed cells were then treated with chloroform, centrifuged at 13,000 × *g* for 15 min, and the upper phase was recovered. Bacterial RNA was subsequently purified using the RNeasy Mini Kit (Qiagen, Germany) following the manufacturer's instructions. RNA concentration and purity were assessed through a spectrophotometer (Eppendorf, Germany).

Subsequently, total bacterial RNA (from 100 ng to 1 μg) was subjected to rRNA removal through the QIAseq FastSelect—5S/16S/23S following the manufacturer's instructions (Qiagen, Germany). The yield of rRNA depletion was checked with a 2200 TapeStation (Agilent Technologies, USA). Then, starting from the rRNA‐depleted samples, a whole transcriptome library was constructed using the TruSeq Stranded mRNA Sample preparation kit (Illumina, San Diego, USA). Finally, samples were loaded on a NextSeq high‐output v2 kit (150 cycles) (Illumina) as indicated by the technical support guide.

The obtained reads were filtered to remove low‐quality reads (minimum mean quality 20, minimum length 150) as well as any remaining ribosomal locus‐encompassing reads using the METAnnotatorX2 pipeline (Milani et al. 2021). The retained reads were aligned to the specific reference genome through Bowtie2 software (Langdon 2015), while the quality of the alignments was evaluated using the Picard software (Dobin et al. 2013). Subsequently, quantification of reads mapped to individual transcripts was achieved through the htseq‐counts script of HTSeq software in “union” mode (Anders et al. 2015). Raw counts were then normalised using CPM (Counts per million mapped reads) for filtering genes with low counts (CPM < 1) and TMM (Trimmed Mean of M‐values) for statistically robust differential gene expression analysis through the EdgeR package (Robinson et al. 2010). Evaluation of the expression differences was calculated for each gene as log_2_ fold change (logFC) of average expression between *B. bifidum* PRL2010 wt and *B. bifidum* PRL2010 *luxS*::pFREM30. Genes showing a logFC > 2.5 or logFC < −2.5, logFC > 1, and a false discovery rate (FDR)‐adjusted *p*‐value < 0.05 were considered significantly differentially expressed.

### 2.9 Mucin‐Based Growth Assay

Overnight cultures of *B. bifidum* PRL2010 wt and *luxS*::pFREM30 strains were washed twice with PBS and then inoculated in freshly prepared mMRS without carbon source, supplemented with mucin at a final concentration of 0.5% (w/v). Cells were inoculated to reach a final OD_600nm_ of ~0.1 and incubated in an anaerobic cabinet. Cell growth was evaluated by monitoring the optical density at 600 nm by using the Stratus microplate reader (Cerillo, USA). Cultures were grown in triplicates, and the resulting growth data sets were expressed as the average of these replicates.

### 2.10 Adhesion of *B. bifidum* PRL2010 Wt and *luxS* ::pFREM30 Strains to HT29‐MTX Cells

Bifidobacterial ability to adhere to HT29‐MTX cells was evaluated as previously described (Rizzo et al. 2023, 2024). In detail, human colon carcinoma‐derived mucin‐secreting goblet HT29‐MTX cells (kindly provided by Prof. Antonietta Baldi, University of Milan) were cultured in Minimum Essential Medium (MEM) with high glucose (4.5 g/L) as previously reported (Bianchi et al. 2019). The medium was supplemented with 10% fetal bovine serum (FBS), 4 mM glutamine, 100 μg/mL streptomycin, 100 U/mL penicillin, and 10 mM HEPES. For the experiment, HT29‐MTX cells were seeded on microscope cover glasses previously settled in 10 cm^2^ Petri dishes. Eukaryotic cells were washed twice with PBS before the addition of bifidobacterial cells.

*B. bifidum* PRL2010 wt and *luxS*::pFREM30 strains were grown in Infant Gut Super Medium (IGSM) (Alessandri et al. 2022) until a concentration of 10^8^ cells/mL was reached. The two strains were then centrifuged at 1000 × *g* for 8 min, resuspended in PBS, and aliquoted on the HT29‐MTX cells. After an incubation of 1 h at 37°C, cells were washed twice with PBS to remove unbound bacteria. Subsequently, cells were fixed with methanol and incubated for 8 min at room temperature. Finally, a Giemsa stain solution (1:20 in PBS) was added to the cells. After an incubation of 30 min with the stain in the dark at room temperature, cells were washed twice with PBS and cover glasses were removed from the Petri plate, mounted on a glass slide, and examined using a Leica DM 1000 phase contrast microscope (objective: 100X/1.4 oil). The number of adherent bacteria was determined by calculating the “adhesion index” [(average number of bacterial cells counted on 10 random spots × number of HT29‐MTX cells)/100], as previously described (Rizzo et al. 2023).

### 2.11 Human Cell Line Culture

Human colorectal adenocarcinoma‐derived Caco‐2 cells (purchased from ATCC) and HT29‐MTX were cultivated in MEM and Dulbecco's Modified Eagle's Medium (DMEM) with high glucose (4.5 g/L) and 10 mM of sodium pyruvate, respectively, as previously described (Bianchi et al. 2019). Both growth media were supplemented with 10% Fetal Bovine Serum (FBS), 2 mM glutamine, 100 μg/mL streptomycin, and 100 U/mL penicillin. Cultures were maintained at 5% CO_2_ at 37°C and passaged three times a week. Subsequently, a mixed suspension of Caco‐2 and HT29‐MTX cells (7:3) was seeded in DMEM + FBS at a density of 10^5^ cells/cm^2^ into cell culture inserts with membrane filters (pore diameter size of 0.4 μm) for Falcon 24‐well multitrays (Betcon, Dickinson & Company, Franklin Lakes, NJ, USA), and cultured for 21 days with a medium replacement every 3 days until a tight monolayer was obtained (TEER > 600 Ω cm^2^).

### 2.12 Human Cell Line Monolayer Exposure to PRL2010 Wt and PRL2010 *luxS* ::pFREM30

After 21 days following seeding, the culture medium of the 24‐well plates was replaced with fresh antibiotic‐free DMEM. Subsequently, PRL2010 wt and PRL2010 *luxS*::pFREM30 were separately added to the Caco‐2/HT29‐MTX cell monolayer at a final concentration of 10^8^ cells/mL, as previously described (Alessandri et al. 2023; Tarracchini et al. 2023). The 24‐well plate was then incubated under aerobic conditions with 5% CO_2_ at 37°C. After 4 h of incubation, bacterial cells and human cell lines were separately recovered in RNA later and preserved at −80°C until processing.

Specifically, for this experiment, *B. bifidum* PRL2010 wt and *B. bifidum* PRL2010 *luxS*::pFREM30 were grown in mMRS broth under anaerobic conditions at 37°C. Once the exponential growth phase was reached, bifidobacterial cells were enumerated through the Thoma cell counting chamber (Herka), diluted to a final concentration of 10^8^ cells/mL, washed twice in PBS, resuspended in 400 μL of fresh antibiotic‐free DMEM, and seeded on Caco‐2/HT29‐MTX cell monolayers. For both strains, the experiment was carried out in triplicate.

### 2.13 Bacterial Gene Expression Analysis by qRT‐PCR

Quantitative real‐time reverse transcription‐PCR (qRT‐PCR) primers (see Table S1) were used to amplify BBPR_RS00165, BBPR_RS05740, BBPR_RS08700, BBPR_RS08925, BBPR_RS04805, BBPR_RS09110, BBPR_RS06690 genes of *B. bifidum* PRL2010. Primer design criteria were based on amplicon size of approximately 100 bp and melting temperatures between 58°C and 64°C, and a qRT‐PCR was performed using the CFX96 system (Bio‐Rad, CA). PCR products were detected with SYBR green fluorescent dye and were amplified using the following protocol: one cycle of 95°C for 2 min, followed by 40 cycles of 95°C for 15 s and 60°C for 1 min. A melting curve was obtained by using temperatures of 65°C to 95°C that increased at a rate of 0.5°C/s.

Each PCR mixture contained 7.5 μL 2× SYBR green SuperMix (Thermofisher, USA), 5 μL of diluted cDNA, the forward and reverse primers each at a concentration of 0.33 μM, and enough nuclease‐free water so that the final volume was 15 μL. In each run, negative controls (no cDNA) for each primer set were included.

### 2.14 Eukaryotic Gene Expression Analysis Through qRT‐PCR

To evaluate whether the presence of a functional *luxS* gene may have a role in modulating the expression of certain eukaryotic genes following 4 h exposure to *B. bifidum* PRL2010 wt and *B. bifidum* PRL2010 *luxS*::pFREM30, human cells were subjected to RNA extraction as previously described (Alessandri et al. 2023; Tarracchini et al. 2023). Briefly, total RNA from human cell monolayers was extracted by adding 350 μL of RLT buffer from the RNeasy Mini Kit (Qiagen, Germany) and then following the manufacturer's instructions. Subsequently, reverse transcription to cDNA was achieved with the iScript Select cDNA synthesis kit (Bio‐Rad Laboratories) using the following thermal cycle: 5 min at 25°C, 30 min at 42°C, and 5 min at 85°C. The mRNA expression levels related to genes encoding for cytokines, tight junctions, and genes involved in mucin production were investigated with SYBR Green technology by means of a quantitative RT‐PCR (qRT‐PCR) using the PowerUp SYBR Green Master Mix (Thermo Fisher Scientific, USA) on a Bio‐Rad CFX96 system according to the manufacturer's instructions. PCR products were detected with SYBR green fluorescent dye and amplified with the following protocol: one cycle at 50°C for 2 min, one cycle at 95°C for 2 min, followed by 40 cycles at 95°C for 15 s and Tm (°C) for 1 min. The melting temperature used for each primer pair as well as the primers employed in this study are reported in Table S1. In each plate, negative controls (no DNA) were included for each primer pair. Furthermore, gene expression was normalised to housekeeping genes coding for beta‐globin, glyceraldehyde‐3‐phosphate dehydrogenase, and ribosomal protein L15 (RPL‐15). All results regarding mRNA expression levels were reported as fold‐induction in comparison with the control; that is, Caco‐2/HT29‐MTX cells not exposed to any bifidobacterial strains.

### 2.15 Impact of Exposure to AI‐2 Produced by Other Bifidobacteria on *B. bifidum* PRL2010 *luxS* ::pFREM30 Gene Expression

To evaluate whether the presence of AI‐2 released by other bifidobacteria can be exploited by the mutant strain to restore the metabolic/physiological functions affected by the inability to produce this signalling molecule, *B. bifidum* PRL2010 *luxS*::pFREM30 was exposed to all the metabolites, including AI‐2, produced by two other bifidobacterial strains; that is, *B. bifidum* PRL2010 wt and *Bifidobacterium breve* 12 L. For this purpose, the Cerillo Co‐Culture Duet System was employed (Cerillo, USA). This system allows two bacterial strains to be physically separated thanks to the presence of a porous membrane (pore size of 0.2 μm), while still ensuring a fluid exchange, including metabolites, between the two cultivated strains. In detail, the three considered bifidobacterial strains were grown overnight independently in a growth medium simulating the human gut environment; that is, IGSM (Alessandri et al. 2022) at 37°C under anaerobic conditions. Subsequently, the cells were enumerated by using a Thoma cell counting chamber (Herka), diluted to reach a final concentration of 10^8^ cells/mL, washed in PBS, and resuspended in fresh IGSM. Subsequently, 800 μL of each bifidobacterial strain was aliquoted into the Cerillo Co‐Culture Duet System to establish a fluidic contact between *B. bifidum* PRL2010 *luxS*::pFREM30 and the wt strain as well as between the mutant strain and *B. breve* 12 L. Cells were incubated at 37°C under anaerobic conditions for 8 h to prevent RNA degradation. *B. bifidum* PRL2010 *luxS*::pFREM30, not exposed to any other strains, was used as a control sample by culturing it in the same system, without the membrane separating the two duet chambers. After incubation, cells were recovered through centrifugation at 5000 × *g* for 5 min and stored at −80°C until RNA extraction. RNA extraction and sequencing were performed as above described. All experiments were carried out in triplicates.

### 2.16 Statistical Analysis

SPSS software was used to compute statistical analysis; that is, the Shapiro–Wilk test, Student's *t*‐test, Mann–Whitney *U* test, and ANOVA. In detail, to select between parametric and non‐parametric tests, before performing statistical analyses, a Shapiro–Wilk test was used to evaluate data normality.

## 3 Results and Discussion

### 3.1 Genomic Survey of *luxS* Gene Among Members of the *Bifidobacterium* Genus

**FIGURE 1:**
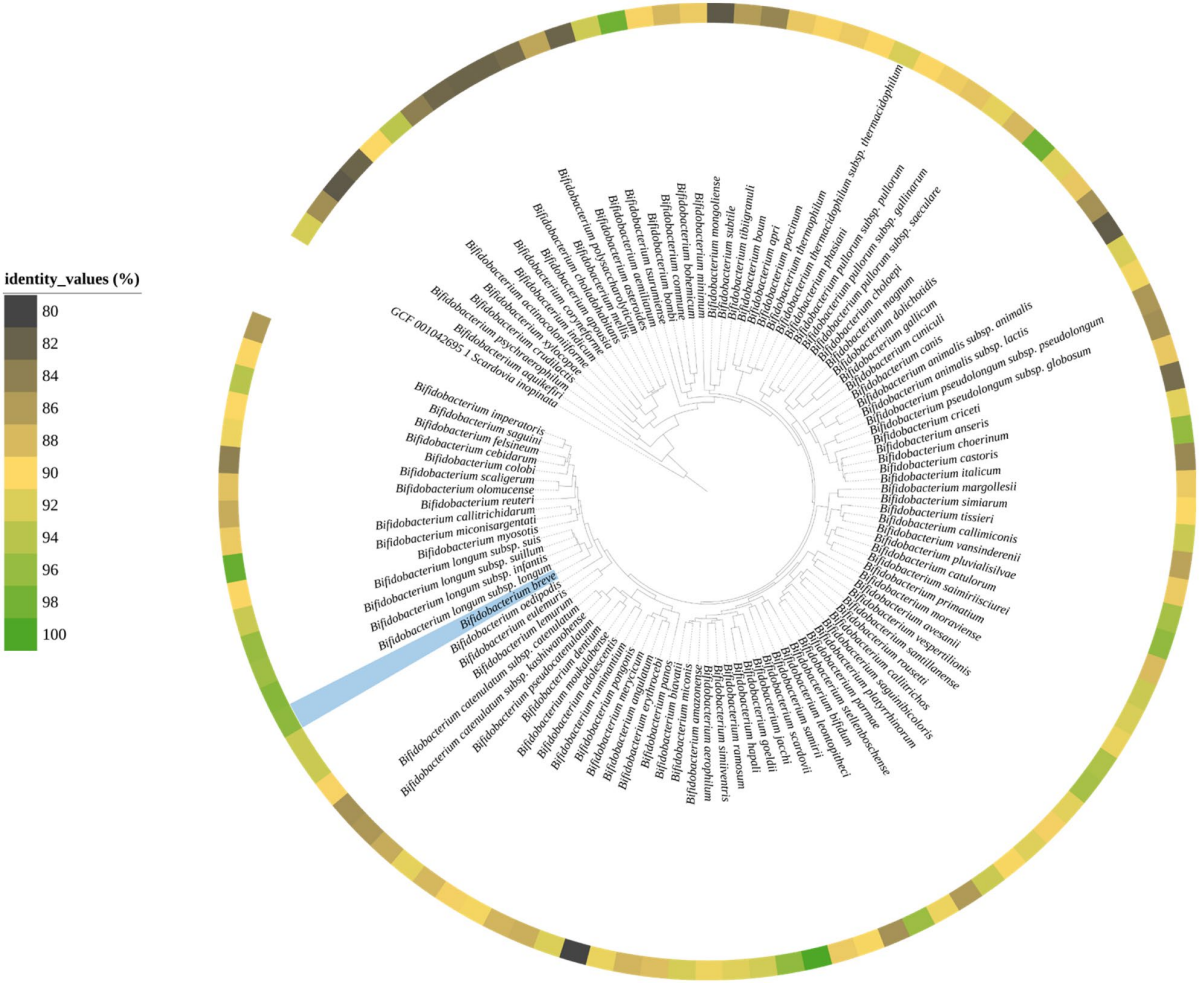
Distribution and conservation of *lux*S gene across the *Bifidobacterium* genus. The image displays the distribution and sequence identity of *luxS* homologues across the genomes of the bifidobacterial type strains of all the species of the genus *Bifidobacterium*. Percentage identities are calculated relative to the *luxS* gene sequence of *B. breve* UCC2003.

An in silico analysis was performed in order to evaluate the prevalence of homologues of *luxS*, encoding the key enzyme to produce the signal molecule autoinducer‐2 (Christiaen et al. 2014; Sun et al. 2014; Song et al. 2018), among bifidobacterial genomes. Specifically, a BlastP analysis was carried out between the amino acid sequence deduced from the *luxS* homologue previously identified in *Bifidobacterium breve* UCC2003 (Christiaen et al. 2014) and amino acid sequences derived from genes identified in publicly available bifidobacterial genomes present in the RefSeq database from NCBI. Interestingly, in all 3359 analysed bifidobacterial genomes, a *luxS* homologue was detected with high sequence homology (> 80%) (Figure 1), except for 18 bifidobacterial genomes that showed a reduced sequence homology ranging from 74.62% to 79.87%, with respect to the deduced amino acid sequence encoded by *luxS* of *B. breve* UCC2003 (Table S2). Interestingly, 12 of the latter genomes belonged to bifidobacterial species typically found in raw milk or fermented raw milk products, including *Bifidobacterium crudilactis*, *Bifidobacterium mongoliense* , and *Bifidobacterium tibiigranuli* (Figure 1). This finding shows that strains of these species possess a less conserved *luxS* sequence when compared to the input sequence, consistent with a previously reported observation that these species are among the most phylogenetically distant from *B. breve* (Duranti et al. 2020; Lugli et al. 2021; Olofsson et al. 2023). Gene context analysis of *lux*S in representative strains revealed a partially conserved genomic organisation, with only the immediate neighbouring genes well conserved regardless of the degree of *lux*S sequence identity, suggesting that local synteny is more readily maintained in strains with higher *lux*S homology (Figure S1).

These findings not only highlight that the *luxS* gene is conserved in the genomes of all currently known bifidobacterial taxa, confirming what has previously been observed for a much smaller number of genomes and bifidobacterial species (Christiaen et al. 2014; Sun et al. 2014), but also suggest that all bifidobacteria are capable of producing this signalling molecule. In this context, to better characterise the role played by *luxS* in the species *B. bifidum* , in vitro experiments were performed using the prototype human gut‐derived *B. bifidum* PRL2010 (Rizzo et al. 2024).

### 3.2 Detection of AI‐2 Like Activity

**FIGURE 2:**
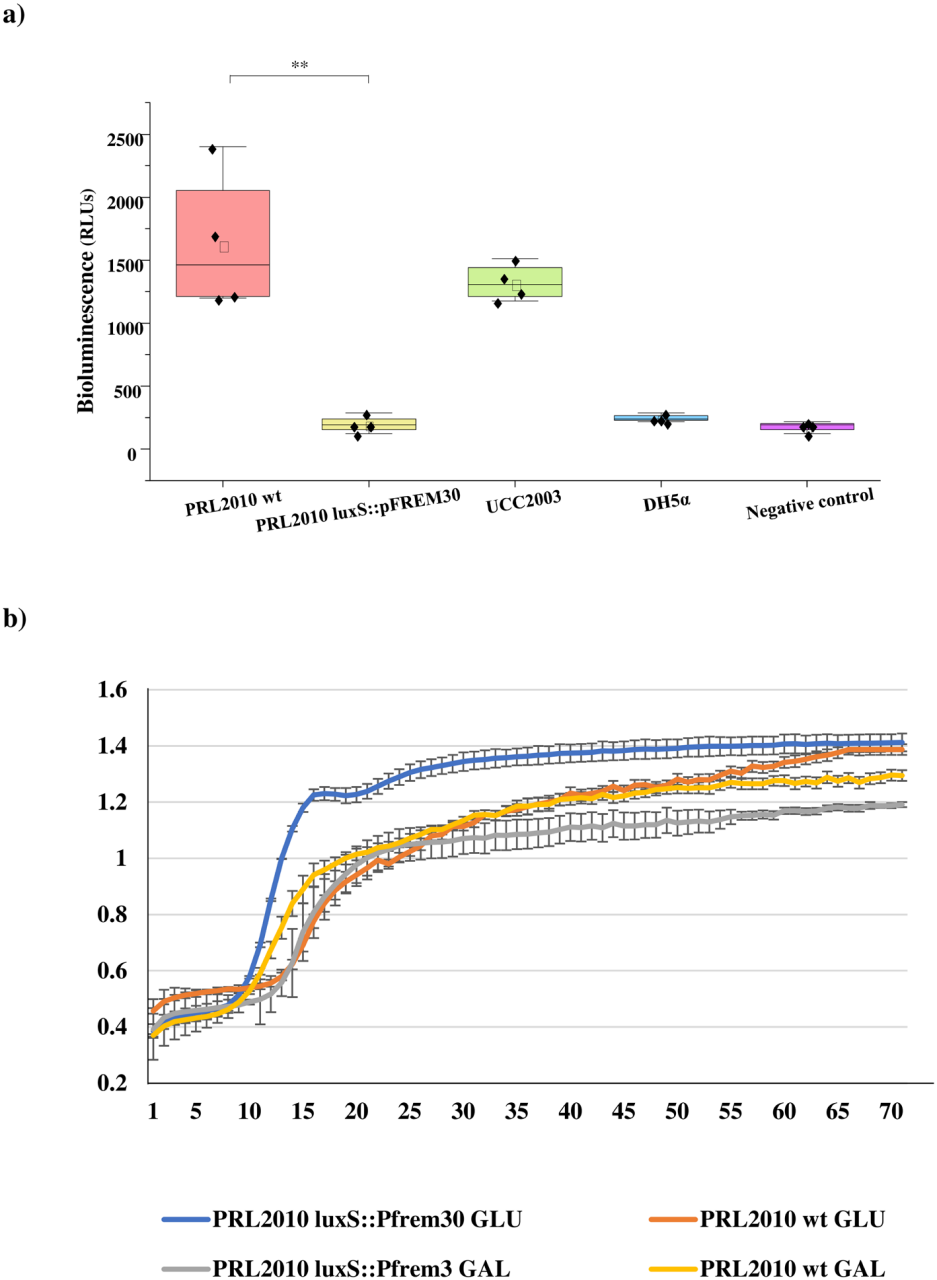
Impact of *luxS* inactivation on *B. bifidum* PRL2010. (a) Depicts the box and whisker plot of the recorded bioluminescence signal produced by the *V. harveyi* BB170 biosensor strain when in the presence of sterile and neutralised supernatant of *B. bifidum* PRL2010 wt, *B. bifidum* PRL2010 *luxS*::PFREM30, *B. breve* UCC2003 (positive control), *E. coli* DH5α (negative control), or a medium‐only control (negative control). The x‐axis reports the considered supernatants, while the *y*‐axis shows the detected bioluminescence expressed as Relative Luminescence Units (RLUs). Boxes are determined by the maximum and minimum values and correspond to the box extreme values. Lines inside the boxes represent the average, while the squares correspond to the median. **: *p*‐value < 0.01. (b) Shows the growth curves of *B. bifidum* PRL2010 wild‐type and relative mutant *luxS*::PFREM30 grown in a medium containing glucose (GLU) or galactose (GAL) as the sole carbon source with standard deviation. Growth was measured at OD600.

To evaluate the in vitro impact of a mutated *luxS* on *B. bifidum* PRL2010, its isogenic *luxS*‐insertion mutant was obtained, here designated as *B. bifidum* PRL2010 *luxS*::pFREM30, following a previously described protocol (Rizzo et al. 2024). We then assessed the effect of the insertional mutation on AI‐2 production, employing an AI‐2 activity detection assay with *Vibrio harveyi* BB170 as a reporter strain, as previously described (Christiaen et al. 2014; Sun et al. 2014). As expected, inactivation of *luxS* in *B. bifidum* PRL2010 *luxS*::pFREM30 causes a drastic and significant decrease in AI‐2 production (ANOVA *p*‐value < 0.01) when compared to the wt strain, eliciting average levels of bioluminescence of 198 ± 69 and 1632 ± 562 Relative Luminescence Units (RLUs), respectively (Figure 2a). Indeed, *B. bifidum* PRL2010 *luxS*::pFREM30 elicited levels of bioluminescence in *Vibrio harveyi* BB170 that are similar to those produced by this reporter strain in response to the supernatant of the AI‐2 negative control strain *E. coli* DH5α or for the negative control (only growth medium) (ANOVA *p*‐value > 0.05) (Figure 2a). Conversely, bioluminescence values recorded for the wt strain were similar to those obtained for another bifidobacterial strain whose ability to produce AI‐2 had previously been described; that is, *B. breve* UCC2003 (1326 ± 147 RLUs) (ANOVA *p*‐value > 0.05) (Figure 2a) (Christiaen et al. 2014). Thus, this data not only underlines the crucial role played by *luxS* in AI‐2 production in the wt strain *B. bifidum* PRL2010 but also demonstrates the inability of the isogenic mutant strain to produce AI‐2 and consequently to regulate AI‐2 mediated physiological processes.

### 3.3 Genomic and Phenotypic Characterisation of *B. bifidum* PRL2010 *luxS* ‐Insertion Mutant

The reconstructed genome after long and short read sequencing was compared with that of *B. bifidum* PRL2010 wt strain, revealing the insertion of 2090 bp of the plasmid DNA in conjunction with the expected duplication of an internal fragment of the *luxS* gene sequence, confirming the homologous recombination within the *luxS* gene sequence. Furthermore, the comparison of genome sequences did not reveal other genomic modifications, demonstrating that the insertion of the plasmid DNA in the lux*S* gene sequence was the only identifiable genetic difference between the mutant and the wt chromosomal structure, thus confirming that there were no additional genomic alterations following the strain manipulation.

In addition, the stability of the integrated pFREM30 plasmid used to establish the *B. bifidum* PRL2010 *luxS*::pFREM30 was assessed by performing a mutant stability assay. In detail, *B. bifidum* PRL2010 *luxS*::pFREM30 was cultivated in the presence or absence of 5 μg/mL chloramphenicol and sub‐cultured daily for a total of 2 weeks in both mMRS and IGSM media. Furthermore, every 2 days of sub‐culturing, cells were spotted on mMRS plates with or without chloramphenicol, and after 48 h of incubation, the presence of the integrated plasmid was assessed through colony PCR, as previously described (Rizzo et al. 2024). Interestingly, this assay not only confirmed the stability of the integrated plasmid in the mutant over time in the absence of antibiotic selection, but also that the colony‐forming unit (CFU) number remained stable (10^8^ CFU/mL) for the full duration of the experiment in all tested conditions. Based on this result and to avoid introducing a variable that could negatively influence growth assays, all downstream experiments were performed without the addition of any antibiotics to the culture medium.

Finally, since it has previously been reported that LuxS plays an important role in bacterial central metabolism and physiology participating in the activated methyl cycle (Xavier and Bassler 2003; Vendeville et al. 2005; Parveen and Cornell 2011; Christiaen et al. 2014), growth assays were performed using monosaccharides as carbon sources in order to assess if the insertional mutant *B. bifidum* PRL2010 *luxS*::pFREM30 elicited growth performances different from the wt strain. In detail, glucose and galactose were chosen as monosaccharides to be tested since they represent two carbon sources on which PRL2010 wt grows reaching high yields (Turroni, Strati, et al. 2012). Notably, *B. bifidum* PRL2010 wt and *luxS*::pFREM30 strains grew with similar performances on both these carbon sources (Student's T test *p*‐value > 0.05 in both cases). Indeed, OD_600nm_ values of 1.322 and 1.429 on glucose as well as of 1.247 and 1.135 on galactose were recorded for wt and *luxS*::pFREM30 strains, respectively (Figure 2b). These findings indicate that the mutation does not significantly impact the growth performance of the strain when cultivated on monosaccharides as the only carbon source.

### 3.4 Effects of *LuxS* Inactivation on Gene Expression in *B. bifidum* PRL2010

**FIGURE 3:**
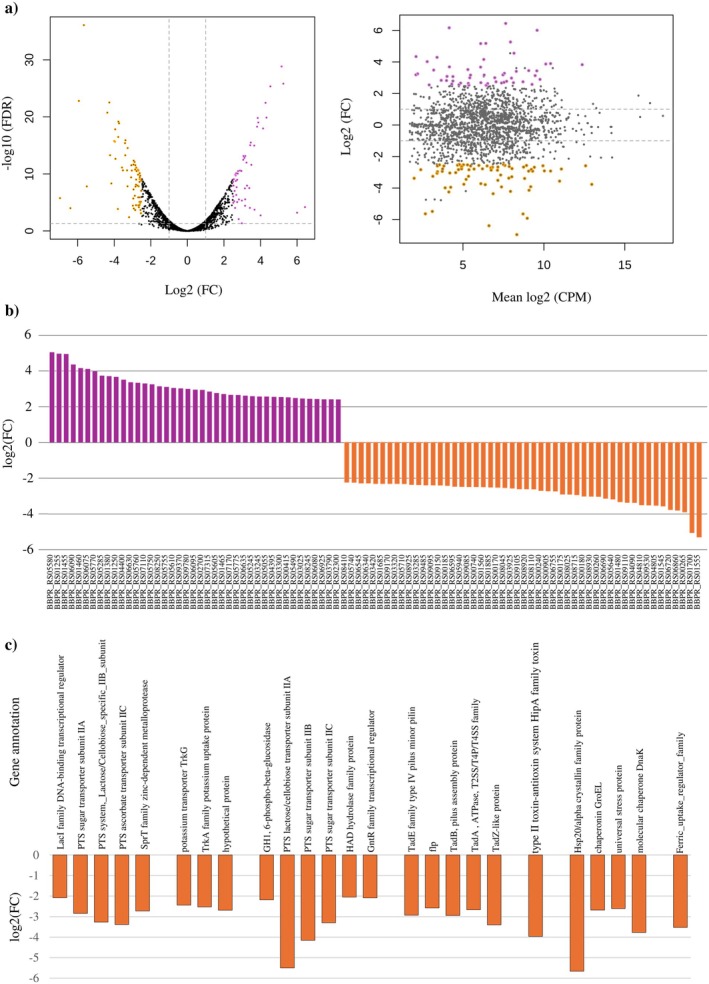
Differential gene expression analysis of *B. bifidum* PRL2010 *luxS*::PFREM30 versus *B. bifidum* PRL2010. (a) Depicts Volcano plot (left) and MA plot (right) showing differential expression between the mutant and wild‐type strains. Each point represents a gene, with significantly up‐regulated genes (log_2_FC > 2.5, FDR < 0.05) in violet and down‐regulated genes (log_2_FC < −2.5, FDR < 0.05) in orange. (b) Shows an overview of the differentially expressed genes sorted by log_2_ fold change. Violet bars indicate significantly up‐regulated genes, orange bars denote down‐regulated ones. (c) Highlights functional categories such as carbohydrate phosphotransferase systems (PTSs), pilus proteins, as well as potassium and iron transporters, showing substantial repression in the mutant strain.

To investigate the impact of *luxS* gene disruption on *B. bifidum* PRL2010 gene transcription, *B. bifidum* PRL2010 wt and *luxS*::pFREM30 strains were grown individually in a growth medium simulating the human gut (Alessandri et al. 2022). Specifically, only genes showing a log_2_ fold‐change ≥ 2.5 or ≤ −2.5 coupled with an FDR‐adjusted *p*‐value ≤ 0.05 were considered as significantly differentially expressed between the two strains in the RNA sequencing experiment (Figure 3a). In this context, 53 genes (2.79% of the total number of genes encompassing the chromosome of *B. bifidum* PRL2010) were shown to be significantly up‐regulated, and 71 genes down‐regulated (3.74% of the total number of genes of the genome sequences of PRL2010) in the insertion mutant strain when compared to the wt (Figure 3b; Table 1; Table S3). Looking in detail, two distinct genetic loci consisting of five and six adjacent genes, respectively, all predicted to encode phosphoenolpyruvate phosphotransferase systems (PEP‐PTSs); used for carbohydrate uptake with concomitant phosphorylation (Deutscher et al. 2006) were shown to be completely down‐regulated in the *luxS*::pFREM30 mutant when compared to the wt strain (Figure 3c; Table 1; Table S3). In addition, significant reduction in the number of transcripts of three adjacent genes putatively involved in potassium uptake was observed (Figure 3c; Table 1; Table S3). Beyond carbohydrate and cation transport, the *luxS* mutation was shown to promote a significant transcriptional reduction of all the genes of the *tad* locus, which is responsible for producing tight adherence pili, except *tadF* and *tadV* (Figure 3c; Table 1; Table S3) (O'Connell Motherway et al. 2011). These findings suggest that lux*S* plays a role in regulating the uptake of particular substances; that is, certain cations and carbohydrates, and the expression of extracellular structures; that is, Tad pili affecting bifidobacterial ability to adhere to the human host, strengthening what has previously been observed for the genus *Lactobacillus* and other bifidobacterial strains (Lebeer et al. 2007; Buck et al. 2009; Wilson et al. 2012; Christiaen et al. 2014). In addition, one gene involved in a so‐called toxin‐antitoxin system was significantly down‐regulated in the *luxS*::pFREM30 strain; that is, BBPR_RS04805 (Figure 3c; Table 1; Table S3). In this context, since the latter systems have been described to participate in various processes, including biofilm formation, bacterial persistence, and antibiotic or stress tolerance, the down‐regulation of these genes in the insertion mutant strain strengthens the notion that the inability to produce AI‐2 affects the ability of *B. bifidum* PRL2010 to colonise the human gut (Christiaen et al. 2014; Sun et al. 2014; Deng et al. 2022; Li et al. 2022; Hu et al. 2024). Finally, the presence of a functional *luxS* seemed to be crucial to allow *B. bifidum* PRL2010 to activate a prompt response to stress conditions, including heat, osmotic, and oxidative stress. Indeed, a significant down‐regulation of four genes predicted to be involved in stress response was observed in the wt strain when compared to PRL2010 *luxS*::pFREM30 (Figure 3c; Table 1; Table S3). In addition, as observed for *B. breve* UCC2003 (Christiaen et al. 2014), also in *B. bifidum* PRL2010, a disrupted *luxS* induced a down‐regulation of a gene encoding a predicted ferric uptake regulator, indicating that the *luxS* mutation may affect the insertion mutant's ability to regulate the acquisition of iron. qRT‐PCR assays were also performed on a subset of genes to confirm the transcriptomic data (Table S1; Figure S2).

**TABLE 1:** Relative normalised gene expression levels of genes described in the text expressed in vitro in lux*S*::PFREM30 mutant with respect to the *B. bifidum* PRL2020 wild type.

Locus tag	Logfc	*p*	Annotation
BBPR_RS00165	−2.076	2.64E‐08	LacI family DNA‐binding transcriptional regulator
BBPR_RS00170	−2.845	6.53E‐14	PTS sugar transporter subunit IIA
BBPR_RS00175	−3.264	4.29E‐17	PTS system_Lactose/Cellobiose_specific_IIB_subunit
BBPR_RS00180	−3.384	1.33E‐18	PTS ascorbate transporter subunit IIC
BBPR_RS00185	−2.727	5.69E‐10	SprT family zinc‐dependent metalloprotease
BBPR_RS05735	−2.439	1.34E‐09	Potassium transporter TrkG
BBPR_RS05740	−2.524	6.26E‐09	TrkA family potassium uptake protein
BBPR_RS05745	−2.684	4.36E‐06	Hypothetical protein
BBPR_RS08700	−2.179	2.45E‐07	GH1, 6‐phospho‐beta‐glucosidase
BBPR_RS08705	−5.499	8.78E‐10	PTS lactose/cellobiose transporter subunit IIA
BBPR_RS08710	−4.154	2.27E‐10	PTS sugar transporter subunit IIB
BBPR_RS08715	−3.298	6.12E‐13	PTS sugar transporter subunit IIC
BBPR_RS08720	−2.048	3.67E‐06	HAD hydrolase family protein
BBPR_RS08725	−2.088	4.69E‐08	GntR family transcriptional regulator
BBPR_RS08905	−2.928	2.05E‐05	TadE family type IV pilus minor pilin
BBPR_RS08910	−2.579	1.43E‐06	Flp
BBPR_RS08920	−2.942	3.45E‐09	TadB, pilus assembly protein
BBPR_RS08925	−2.667	7.89E‐10	TadA, ATPase, T2SS/T4P/T4SS family
BBPR_RS08930	−3.396	8.63E‐16	TadZ‐like protein
BBPR_RS04805	−3.967	2.59E‐18	Type II toxin‐antitoxin system HipA family toxin
BBPR_RS01700	−5.660	4.54E‐40	Hsp20/alpha crystallin family protein
BBPR_RS03285	−2.678	6.85E‐13	Chaperonin GroEL
BBPR_RS03320	−2.610	2.86E‐11	Universal stress protein
BBPR_RS09110	−3.775	4.65E‐22	Molecular chaperone DnaK
BBPR_RS06690	−3.525	6.20E‐13	Ferric_uptake_regulator_family

Overall, these results suggest that the lack of a functional *luxS* gene not only affects PRL2010 transport systems but also appears to undermine its ability to adhere/interact with the host, to respond promptly to stress conditions and to affect potassium transport and iron acquisition.

### 3.5 Impact of a Nonfunctional *lux*S on *B. bifidum* PRL2010 Ability to Grow on and Adhere to Mucin

Members of the genus *Bifidobacterium* possess, in their genetic arsenal, various genes implied in the adhesion to/interaction with the human intestinal epithelium which, overall, assist bifidobacteria in long‐term colonisation of/survival in the extremely competitive gut environment (Alessandri, Milani, et al. 2019; Alessandri et al. 2021; Engevik et al. 2021; Luo et al. 2021; Rizzo et al. 2024). In this context, in vitro adhesion experiments were performed to validate the RNA‐based data revealing that various genes responsible for adhesion to/interaction with the host were affected by LuxS.

**FIGURE 4:**
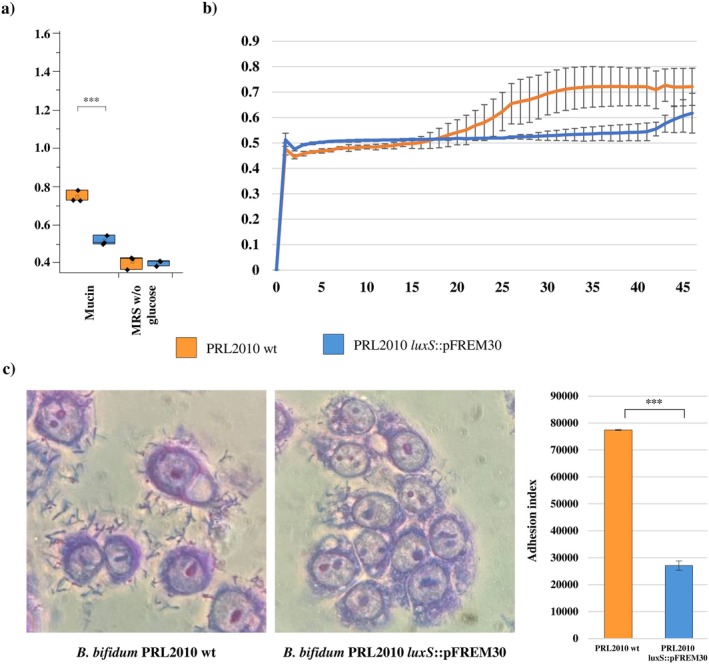
Impact of a nonfunctional *lux*S on *B. bifidum* PRL2010's ability to grow on and adhere to mucin. (a) Shows the box and whisker plot relative to the OD_600nm_ measurement obtained after a 48 h growth of the wt and *luxS*::PFREM30 strains on mucin. In the box and whisker plots, the *y*‐axis shows the OD_600nm_ values. Boxes are determined by the maximum and minimum values and correspond to the box extreme values. Lines inside the boxes represent the average, while the squares correspond to the median. (b) Depicts the growth curves (OD 600 nm) of *B. bifidum* PRL2010 and *luxS*::PFREM30 mutant strains with mucin as the sole carbon source. Cells were grown in biologically independent triplicates. (c) Reports on the left the light microscope images of HT29‐MTX after exposure to either *B. bifidum* PRL2010 wt or *B. bifidum* PRL2010 *luxS*::PFREM30 and coloured with the Giemsa staining. Bar, 10 μm. On the right, the bar plot reports the ability of *B. bifidum* PRL2010 and *B. bifidum* PRL2010 *luxS*::PFREM30 to adhere to HT29‐MTX expressed through the calculation of the adhesion index. Three biological replicates were obtained for each considered strain.

Specifically, to first assess whether a nonfunctional *luxS* gene may play a role in affecting *B. bifidum* PRL2010's ability to degrade and, therefore, retrieve energy from mucin, a growth assay was carried out, using mucin as the sole carbon source. Interestingly, significantly higher growth performances were recorded for *B. bifidum* PRL2010 wt when compared with the mutant strain (Student's *t* test *p*‐value < 0.01), with an OD_600nm_ average of 0.756 and 0.508, respectively (Figure 4a,b). This result indicates that the presence of LuxS plays a relevant role in promoting *B. bifidum* PRL2010's ability to use mucin as a growth substrate.

Furthermore, to investigate whether an inactivated *luxS* gene interferes with the known ability of *B. bifidum* PRL2010 to adhere to/interact with the human intestinal epithelium (Turroni et al. 2010; Serafini et al. 2013; Rizzo et al. 2024), the ability of *B. bifidum* PRL2010 wt and *luxS*::pFREM30 strains to adhere to human mucin‐secreting HT29‐MTX cells was assessed. Interestingly, a statistically significant reduction in the adhesion ability of the mutant strain was recorded when compared to the wt, with an adhesion index of 27,111 ± 1710 and 77,444 ± 192.45, respectively (Mann–Whitney *U* test *p*‐value = 0.046) (Figure 4c). These data suggest that the disruption of *luxS* reduces the ability of PRL2010 to adhere to the intestinal epithelium, possibly undermining the ability of the strain to persist in the intestinal environment.

### 3.6 Impact of AI‐2 Exogenously Produced by Other Bifidobacteria on *B. bifidum* PRL2010 *lux*S::pFREM30

To evaluate whether the AI‐2 produced by other bifidobacteria can be favourably exploited by the mutant to restore those metabolic and physiological activities that are under the control of the signalling molecule, the insertional mutant strain was exposed to the metabolites, expected to include AI‐2, produced by either *B. bifidum* PRL2010 wt or *B. breve* 12 L by using the Cerillo Co‐Culture Duet System. Specifically, this system allows metabolite exchange through fluidic contact yet avoids direct physical interaction between bifidobacterial strains thanks to the presence of a porous membrane. After a fluidic contact of 8 h, *B. bifidum* PRL2010 *lux*S::pFREM30 cells were harvested and subjected to RNA extraction and sequencing (PRL2010 *lux*S::pFREM30 grown individually was used as control sample). Significantly differentially expressed genes were identified as above reported for RNAseq analysis. The supernatant obtained from the growth of this strain was subjected to the AI‐2 like activity detection assay, as above described, and bioluminescence results indeed verified AI‐2 production at a level similar to that above obtained for PRL2010 wt and *B. breve* UCC2003 (ANOVA *p*‐value > 0.05 for both cases), recording an average bioluminescence of 1716 ± 331 RLUs, confirming the ability of this strain to produce AI‐2.

Interestingly, RNAseq data highlighted that only 38 and 1 genes were differentially expressed (showing a log_2_ fold‐change ≥ 2.5 or ≤ −2.5 coupled with a FDR‐adjusted *p*‐value ≤ 0.05) in *B. bifidum* PRL2010 *luxS*::pFREM30 exposed (but not in contact) to *B. breve* 12 L and PRL2010 wt respectively, when compared to the control; that is, *B. bifidum luxS*::pFREM30 grown without any exposure to other strains (Figure S3; Table S1). Interestingly, no genes were found to be markedly down‐regulated, and this result leads to the hypothesis that the mutant may be able to advantageously exploit the AI‐2 produced by other bifidobacteria to regulate gene expression of those genetic sequences that are under AI‐2 control, restoring the metabolic/physiological functions typical of the wt strain. However, further experiments are necessary to validate this hypothesis.

### 3.7 Evaluation of Host Response After Exposure to *B. bifidum* PRL2010 *lux*S::pFREM30

To evaluate whether the lack of a functional *luxS* induces a differential response in the intestinal epithelial cells, a Caco‐2/HT29‐MTX cell monolayer was exposed to the wt or *lux*S::pFREM30 strains for 4 h. Subsequently, eukaryotic cells were subjected to RNA extraction and the expression of a specific set of genes was assessed through qRT‐PCR by considering Caco‐2/HT29‐MTX cells not exposed to bifidobacterial strains as control samples. Specifically, the expression levels of genes responsible for mucus layer production, implicated in maintaining the integrity and homeostasis of the intestinal epithelial barrier, were analysed. In parallel, to evaluate whether the inflammatory response of human cells can change in the absence of a functional *luxS*, transcription levels of genes encoding different cytokines were investigated.

**FIGURE 5:**
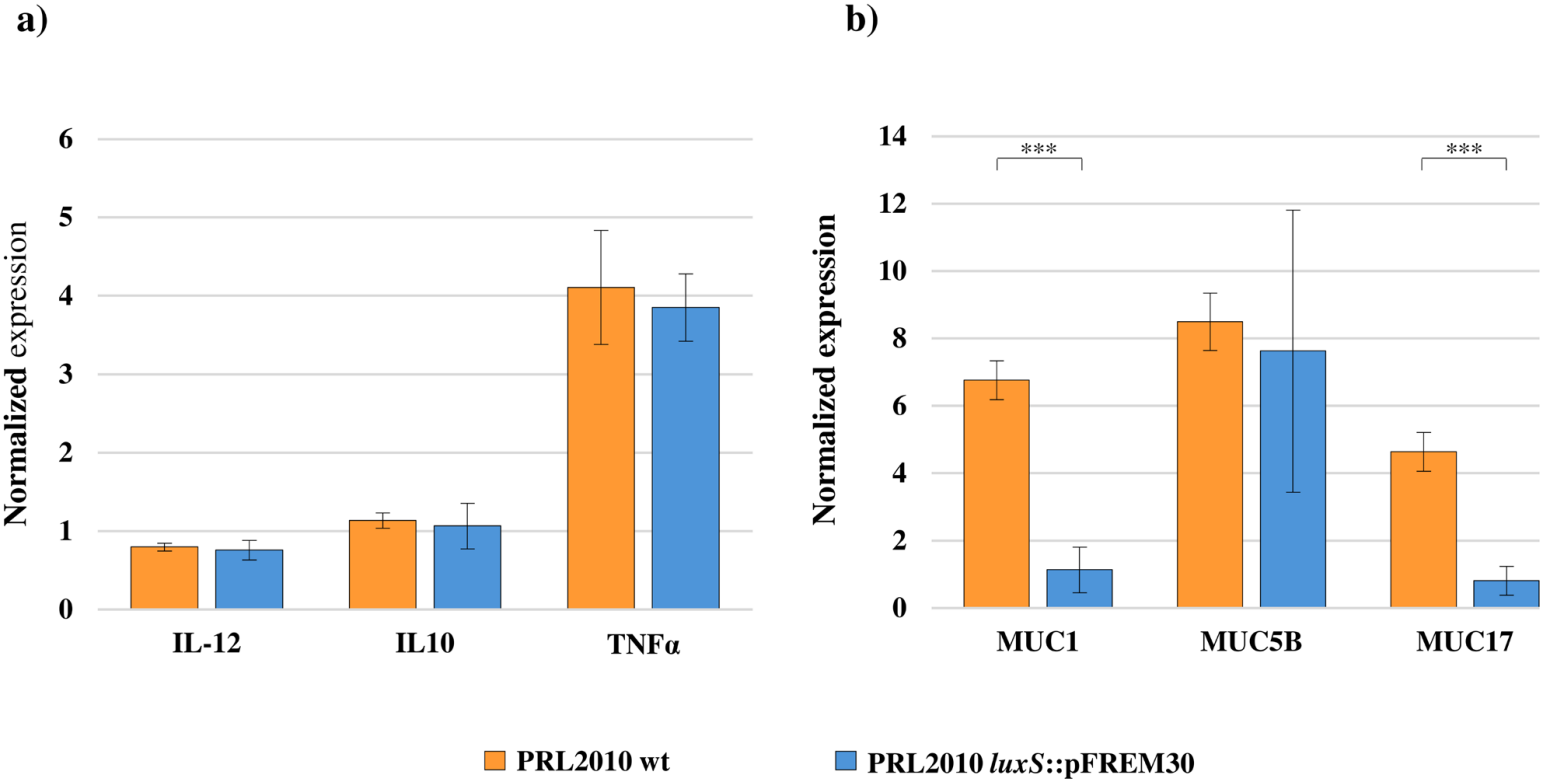
Analysis of host response after exposure to *B. bifidum* PRL2010 *luxS*::PFREM30. (a) Displays RT‐PCR data associated with the gene expression of genes involved in mucin production. (b) Depicts RT‐PCR data linked with the gene expression of cytokines. For both panels, RT experiments were carried out on the Caco2/HT29‐MTX human cell line monolayer after a 4 h exposure to either *B. bifidum* PRL2010 wt or *B. bifidum* PRL2010 *luxS*::PFREM30. The *y*‐axis represents the normalised expression level (ΔCt) according to CFX96 Bio‐Rad software relative to the control (Caco‐2/HT29‐MTX without bifidobacteria). ***: *p*‐value < 0.001.

Interestingly, while the wt strain induced a 4‐ to 8‐fold increase in transcription levels of three tested genes involved in mucus production; that is, *MUC1*, *MUC5B*, and *MUC17*, with respect to the control, the exposure of the human cell line monolayer to PRL2010 *luxS*::pFREM30 caused an appreciably higher expression of the *MUC5B* gene only (Figure 5a). Accordingly, while no significant difference was observed in the transcription levels of the latter gene between the Caco‐2/HT29‐MTX monolayers exposed to the two different strains (Student's *t*‐test *p*‐value = 0.854), a significantly higher expression of *MUC1* and *MUC17* was recorded in the human cell line after contact with the wt when compared to expression levels of these two genes following exposure to the *luxS*::pFREM30 strain (Student's *t*‐test *p*‐value < 0.01 in both cases) (Figure 5a). These results suggest that, since the wt strain possesses an enhanced ability to metabolise mucin as a carbon source, the bacterial‐driven degradation/consumption of this glycoprotein may act as a stimulus to increase mucin production by eukaryotic cells, potentially assisting in maintaining the integrity of the mucus lining the intestinal epithelial wall.

Conversely, with regard to the inflammatory response, no significant differences were recorded in gene expression levels of interleukin‐encoding genes by the Caco‐2/HT29‐MTX cell line when exposed to the wt or *luxS*::pFREM30 strain (Figure 5b). However, even if not significant between the two conditions, human cell lines in contact with the two strains displayed a remarkable induction of the expression levels of the TNF‐α encoding gene (Figure 5b), confirming what has previously been observed. Indeed, bifidobacteria have been depicted as skilled stimulators of TNF‐α, but less effective inducers of other cytokines associated with the systemic immune response (Fink et al. 2007; Okada et al. 2009; Weiss et al. 2010). Therefore, the ability of PRL2010 to play a critical role in alerting the immune system to quickly respond to potential pathogens, yet without triggering systemic inflammation or adverse effects does not seem to be affected by the lack of a functional *luxS*. This suggests that PRL2010 interaction with the host immune system does not appear to be influenced by AI‐2.

## 4 Conclusion

Despite the pioneering role of *Bifidobacterium* species in the colonisation of the human intestinal microbiota and their capacity to persist within this competitive ecosystem throughout the human lifespan, the quorum sensing mechanisms of these commensal microorganisms remain poorly understood (Christiaen et al. 2014). In this context, to elucidate the role of the *luxS* gene—encoding the enzyme responsible for the synthesis of the AI‐2 precursor—an in silico analysis was conducted to assess the distribution of *luxS* homologues across all publicly available bifidobacterial genomes. This survey revealed that *luxS* is both widespread and highly conserved among the analysed strains.

To further investigate the functional implications of *luxS* in *Bifidobacterium bifidum* , an isogenic insertional mutant of the model strain *B. bifidum* PRL2010 was generated. Transcriptomic analysis via RNA‐seq demonstrated that inactivation of *luxS* substantially alters the metabolic profile of the strain. Specifically, the mutant was shown to elicit downregulation of various genes implicated in sugar transport, potassium and iron uptake, and the biosynthesis of extracellular structures such as pili and stress‐response proteins—suggesting a broad regulatory role for *luxS* in host–microbe interaction pathways.

This hypothesis was corroborated by a notable reduction in the mutant's growth performance in mucin‐based growth assays, as well as a significant decrease in its adhesion capacity to mucus‐secreting HT29‐MTX cells compared to the wild type. Interestingly, the difference in gene expression was markedly attenuated when the mutant was co‐cultured with other bifidobacterial strains capable of producing AI‐2, indicating that AI‐2 molecules synthesised by one species can be utilised by others to modulate gene expression.

Furthermore, the assessment of the host‐related effects revealed that disruption of *luxS* does not compromise the established immunomodulatory capacity of PRL2010 to stimulate the host immune system without eliciting a systemic inflammatory response. However, the mutant strain showed a reduced ability to promote mucin production by enterocytes, potentially affecting the integrity of the intestinal mucus layer.

Collectively, these findings demonstrate that loss of a functional *luxS* gene significantly impairs key metabolic and physiological processes in *B. bifidum* PRL2010, potentially limiting its ability to effectively colonise and persist within the human intestinal niche. Nevertheless, this deficiency can be partially mitigated by exogenous AI‐2 signalling from neighbouring bifidobacterial populations. Future studies involving the construction of a genetically complemented strain will be necessary to definitively validate these observations.

## Author Contributions

**Francesca Turroni:** writing – original draft, conceptualization, writing – review and editing, funding acquisition, supervision, data curation, project administration. **Chiara Tarracchini:** formal analysis, software, visualization, writing – review and editing, data curation. **Gabriele Andrea Lugli:** formal analysis, software, writing – review and editing, data curation. **Laura Maria Vergna:** methodology, investigation. **Giulia Alessandri:** methodology, investigation. **Sonia Mirjam Rizzo:** methodology, investigation. **Massimiliano G. Bianchi:** investigation, methodology. **Tom Coenye:** investigation, methodology, writing – review and editing. **Emanuele Selleri:** software, formal analysis. **Ovidio Bussolati:** writing – review and editing. **Douwe van Sinderen:** writing – review and editing. **Marco Ventura:** writing – review and editing, data curation.

## Conflicts of Interest

The authors declare no conflicts of interest.

## Supporting information


**Figure S1:** Genomic context of *lux*S in representative bifidobacterial strains. Gene maps show the *luxS* gene and its flanked regions in six representative bifidobacterial genomes, with the top three showing high sequence similarity to the *lux*S gene of *B. breve* UCC2003, and the bottom three representing strains with reduced homology. Genes were categorized according to their functions with differently coloured arrows.

**Figure S2:** Relative transcription level of seven PRL2010 genes that were differentially expressed in wt and mutant by qPCR. Figure shows transcription levels of BBPR_RS00165, BBPR_RS05740, BBPR_RS08700, BBPR_RS08925, BBPR_RS04805, BBPR_RS09110, BBPR_RS06690 genes in wt and in PRL2010 *luxS*::pFREM30 mutant.

**Figure S3:** Differential gene expression of *B. bifidum* PRL2010 *luxS*::pFREM30 in fluidic contact with *B. breve* or with the wild‐type strain. In panel a, transcriptional changes in *B. bifidum* PRL2010 *luxS*::pFREM30 in fluidic coculture with *B. breve* are shown through Volcano plot (left) and MA plot (right). A subset of genes is significantly up‐regulated (violet; log_2_FC > 2.5, FDR < 0.05). No significant down‐regulation is observed under this condition. Panel b depicts Volcano plot (left) and MA plot (right) comparing the mutant exposed to the metabolites of the wild‐type strain. Very limited differential expression is observed, with only a small number of genes marginally regulated (orange points; log_2_FC < −2.5, FDR < 0.05).

Data S1.

## Data Availability

Raw sequences from RNA sequencing and raw sequences related to the whole‐genome sequence of the mutant through MinION and Illumina platforms are accessible through the Sequence Read Archive (SRA) under the BioProject accession number PRJNA1196324.
